# Pathology of Amyloid-β (Aβ) Peptide Peripheral Clearance in Alzheimer’s Disease

**DOI:** 10.3390/ijms252010964

**Published:** 2024-10-11

**Authors:** Andrey Tsoy, Bauyrzhan Umbayev, Aliya Kassenova, Bibifatima Kaupbayeva, Sholpan Askarova

**Affiliations:** 1 Center for Life Sciences, National Laboratory Astana, Nazarbayev University, Astana 010000, Kazakhstan; andrey.tsoy@nu.edu.kz (A.T.); bauyrzhan.umbayev@nu.edu.kz (B.U.); aliya.kassenova@nu.edu.kz (A.K.); bibifatima.kaupbayeva@nu.edu.kz (B.K.); 2 Faculty of Natural Sciences, Eurasian National University, Astana 010000, Kazakhstan

**Keywords:** Alzheimer’s disease, amyloid-β peptide, peripheral clearance, enzymatic degradation

## Abstract

Although Alzheimer’s disease (AD) is traditionally viewed as a central nervous system disorder driven by the cerebral accumulation of toxic beta-amyloid (Aβ) peptide, new interpretations of the amyloid cascade hypothesis have led to the recognition of the dynamic equilibrium in which Aβ resides and the importance of peripheral Aβ production and degradation in maintaining healthy Aβ levels. Our review sheds light on the critical role of peripheral organs, particularly the liver, in the metabolism and clearance of circulating Aβ. We explore the mechanisms of Aβ transport across the blood–brain barrier (BBB) via transport proteins such as LRP1 and P-glycoprotein. We also examine how peripheral clearance mechanisms, including enzymatic degradation and phagocytic activity, impact Aβ homeostasis. Our review also discusses potential therapeutic strategies targeting peripheral Aβ clearance pathways. By enhancing these pathways, we propose a novel approach to reducing cerebral Aβ burden, potentially slowing AD progression.

## 1. Introduction

In 1970, Glenner et al. published the first report on the amino acid analysis of proteins found in amyloid fibrils. In 1984, George G. Glenner and Caine W. Wong were the first to describe the sequence of the amyloid beta peptide [1]. This pioneering works laid the foundation of the amyloidogenic hypothesis, which proposes that the accumulation of Aβ in the brain parenchyma is a primary factor in the development of Alzheimer’s disease. The toxicity and metabolism of Aβ have since been the main topics of research into the causes and potential treatments for Alzheimer’s disease.

Aβ peptide is a metabolite of amyloid precursor protein (APP), formed through its sequential proteolysis primarily within neurons. APP is synthesized in the endoplasmic reticulum and transported to the cell membrane in vesicles through the Golgi apparatus and the trans-Golgi network. The length of the APP chain may vary, with the most common isoforms containing 695, 751, or 770 amino acids [2]. APP is produced in different parts of the human body, but the predominant isoform in the brain is APP 695, which is strongly linked to AD pathology [2].

APP undergoes two types of sequential proteolytic processing on the cell membrane: a non-amyloidogenic pathway involving cleavage by α-secretase and an amyloidogenic pathway with initial cleavage by β-secretase. In both pathways, further cleavage occurs through γ-secretases. Amyloidogenic processing of the APP by β- and γ-secretase leads to the production of Aβ monomers of different lengths, of which the Aβ_1–40_ is the primary species and the Aβ_1–42_ is the most fibrillogenic and predominant component in AD plaques [3]. Although β-secretase and γ-secretase are the primary enzymes responsible for Aβ production, other enzymes, such as caspases, Meprin β, and cathepsins, can also contribute under specific pathological conditions [4,5,6]. In the brain, Aβ aggregates and forms deposits ranging from soluble oligomeric molecules to larger, more stable amyloid fibrils [7].

In turn, Aβ concentration in the brain is regulated by a few pathways, such as neuronal and glial clearance. Neurons, microglia, and astrocytes utilize similar pathways to internalize Aβ, primarily through receptor-mediated endocytosis, followed by degradation within lysosomes. The low-density lipoprotein receptor-related protein 1 (LRP1) plays a pivotal role in this process, acting as the major receptor for Aβ uptake [8,9,10]. In addition to cellular clearance, other crucial pathways regulate Aβ levels in the brain. These include the transport of Aβ across the BBB into the bloodstream, as well as its clearance via the cerebrospinal fluid (CSF) and the glymphatic system [11,12,13].

Furthermore, Aβ is cleaved from the blood through a few primary pathways: phagocytosis, enzymatic degradation, transport to other organs involved in Aβ catabolism, such as the liver and kidneys, and the influx transport of Aβ from the bloodstream into the brain parenchyma [14,15,16]. Approximately 40–60% of the Aβ synthesized in the brain is degraded by pathways outside the central nervous system [17], and it is now believed that peripheral clearance of Aβ is the most critical in regulating Aβ concentration in the brain parenchyma. Thus, understanding the mechanisms of Aβ metabolism outside of the brain opens avenues for new therapeutic strategies to enhance Aβ clearance and mitigate AD progression.

In this context, the current review explores the mechanisms involved in Aβ metabolism, mainly focusing on its extracerebral clearance. By understanding these pathways, we can identify potential therapeutic targets for Alzheimer’s disease (AD) that could enhance Aβ clearance and slow down the progression of the disease. The review also discusses the role of peripheral organs, such as the liver, in Aβ catabolism. Additionally, the review examines recent advancements in therapeutic strategies designed to improve Aβ clearance, offering insights into how these approaches could be applied to AD treatment.

## 2. Aβ Transport from the Brain to the Periphery

**Figure 1:**
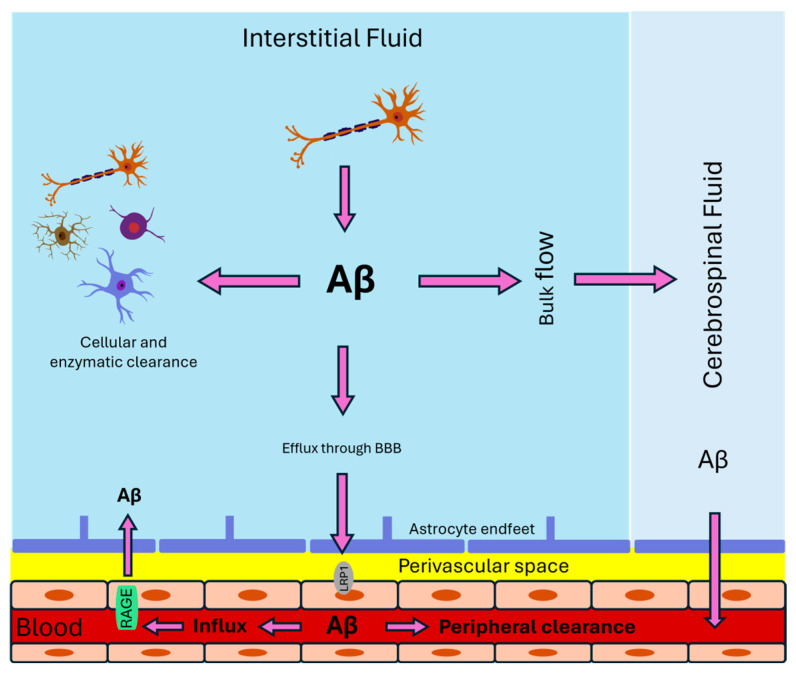
Overview of the key Aβ transport pathways in the brain.

The transport of Aβ from the brain parenchyma to the blood involves several pathways that play critical roles in maintaining brain homeostasis and affecting its pathology. These pathways include the bulk flow via interstitial fluid (ISF) and CSF, as well as receptor-mediated transport across the BBB (Figure 1).

The ISF constitutes approximately 20% of the brain’s volume and plays a crucial role in maintaining homeostasis by regulating ionic balance and facilitating the exchange of nutrients and waste products, including Aβ. CSF enters the interstitial space, mixes with ISF, and facilitates solute exchange via bulk flow [18]. In vivo studies using two-photon imaging have confirmed that ISF proteins, including Aβ, are directly transferred to the CSF through brain parenchyma along the paravascular spaces [19]. Evidence suggests that patients with AD exhibit slower ISF/CSF exchange kinetics, potentially contributing to impaired Aβ clearance [20].

After combining with interstitial fluid, cerebrospinal fluid travels through perivascular spaces and then into paravascular spaces, where it is eventually cleared through the meningeal and cervical lymphatic systems. This process is referred to as the glymphatic pathway, responsible for waste removal. It was demonstrated that fluorescent-tagged Aβ injected into the striatum rapidly moves through CSF and accumulates along the blood vessels. This pathway likely involves aquaporin-4 channels, as evidenced by *AQP4*-null mice showing a 55% reduction in Aβ clearance rates compared to wild-type mice [19]. It is known that glymphatic transport speed depends on the size of molecules [21]. In transgenic APP/PS1 mice, it was confirmed that glymphatic system failure preceded Aβ accumulation, and, in turn, Aβ suppresses glymphatic transport [22].

There is strong evidence that BBB plays an essential role in the homeostasis of Aβ in the brain due to its ability to implement the rapid bidirectional transport of Aβ. In the neurovascular endothelium, various proteins carry out receptor-mediated transport, and the central efflux transport receptor responsible for Aβ clearance is the LRP1 [23,24,25]. LRP1 is a membrane receptor containing two non-covalently bound 85-Kda and 515-Kda chains expressed in many cell types. In brain endothelial cells, this receptor is expressed on the cell membrane’s surface; it also circulates in the blood in soluble LRP1 form (sLRP1) [26]. LRP1 reacts with about 40 ligands, including apolipoproteins and Aβ [27]. This multifunctional receptor plays a crucial role in Aβ endocytosis and cellular signaling in many cell types, and about 50% of Aβ transported to the periphery mediated by LRP1. For instance, this receptor participates in the endocytosis of Aβ in vascular smooth muscle cells [28] and astrocytes [10]; also, this receptor is involved in the metabolism of Aβ in the liver [29].

The mechanisms of Aβ transport from the brain to the bloodstream involving LRP1 as a carrier have been extensively studied in the in vitro BBB model [30]. On the abluminal side of the endothelial cell, Aβ binds to LRP1 and rapidly (less than 30 s) internalizes to the cell in association with PICALM protein, which plays a critical role in clathrin-mediated endocytosis. Intracellular sorting of LRP1/Aβ containing vesicles guided by PICALM protein is a way that vesicles are sorted through early endosomes with further exocytosis on the luminal side of endothelial cells. PICALM preserves Aβ from degradation during intracellular transport by avoiding fusion with lysosomes [30].

If compared with other pathways, receptor-mediated transport is much faster. For example, LRP1 transports Aβ_1–40_ through BBB six times faster than ISF bulk flow. However, the transport intensity of Aβ by LRP1 may depend on other factors such as ApoE and ApoJ [31]. Interestingly, LRP1 may have an opposite role in AD progression. Although LRP1 is one of the critical factors for Aβ clearance, the lack of its expression may shift APP processing towards the non-amyloidogenic pathway and, as a result, reduce the generation of Aβ [32].

Another essential transporter of Aβ is the transmembrane P-glycoprotein (P-gp) [33,34]. P-gp is a well-studied protein expressed on the cell membrane of different types of cells. In the brain, P-gp is expressed in neurons, glial cells, and endothelial cells [35]. P-gp is a 170 kDa ATP-dependent efflux transporter with many transport substrates with different structures. Aβ is much heavier than the largest known P-gp substrate. However, it was shown by molecular dynamic simulation and ATPase activity assay on purified P-gp that Aβ interacts directly with this transport protein [34]. Long before that, Wei et al. demonstrated that P-gp knockout mice crossed with Tg2576 (APP transgenic) showed increased Aβ accumulation [36].

Studies have shown that P-gp expression decreases with age [37]. Aβ itself may suppress P-gp expression through RAGE and NF-κB signaling pathways [38]. It has been demonstrated that the P-gp can directly interact with Aβ, yet additional factors are necessary for P-gp’s activity [34]. In support of this statement, Stork et al. have shown that the main mandatory factors for endosome sorting and rapid Aβ transcytosis are LRP1 associated with P-gp [39]. Unlike LRP1, which is directly responsible for Aβ uptake from the abluminal space [30], P-gp receives Aβ from LRP1 within the endosome. Then, the vesicle containing P-gp/Aβ is sorted through the endosomal network and exocytoses Aβ into the luminal space. During this process, PICALM plays a crucial role similar to that with LRP1, preventing the P-gp/Aβ vesicle from fusing with late endosomes [39].

There is also strong evidence that the receptor for advanced glycation end products (RAGE) is a binding site for Aβ [40,41,42,43,44]. RAGE is a multiligand cell surface receptor typically expressed in brain endothelium and, at low levels, in microglia and neurons [43,44,45]. However, in AD brains, RAGE expression is increased severalfold in cerebral endothelial cells, astrocytes, microglia, and neurons [43,44]. Aβ binding to RAGE has been demonstrated to regulate Aβ transport across BBB, upregulate pro-inflammatory cytokines and adhesion molecules in CECs, and contribute to the transport of Aβ from the cell surface into the intracellular space in cortical neurons [44,46,47]. It has also been shown that RAGE functions as a signal-transducing cell surface receptor for Aβ, and binding of Aβ_1–42_ oligomers to surface RAGE results in the activation of NADPH oxidase to induce ROS generation, and activate downstream pathways, including phosphorylation of ERK1/2 and cPLA_2_ [48].

Some portion of extracellular Aβ in the brain may passively diffuse from the brain parenchyma to the blood across the BBB via the paracellular pathway. Soluble Aβ can diffuse through brain endothelial cell monolayer along a concentration gradient. Tight junction proteins likely play a role in limiting such transport. The highest diffusion rate has been observed in claudin-5 and occludin knockout cells [49]. Additionally, Aβ might autoregulate its non-specific transport by influencing the expression of tight junction proteins. Interestingly, decreased expression of the tight junction proteins claudin-5 and occludin in the cerebral vasculature of transgenic mice (Tg2576) was associated with higher levels of Aβ in the blood but lower levels in the brain and enhanced cognitive function [49].

## 3. Aβ Transport in the Blood

It has been shown that, in the bloodstream, Aβ presented predominantly in bounded forms [50], and the half-life of circulating peripheral Aβ is approximately 2.5–15 min [51,52], while in AD patients, the level of non-bound Aβ is increased up to 3–4 times [53]. Like the LRP1 receptor, sLRP1 is the essential Aβ transporter in the blood. Sagara et al. have shown that sLRP1 typically binds 70% of Aβ_1–40_ and about 90% of Aβ_1–42_ in plasma and may be a critical limitation factor preventing Aβ from influx to the brain parenchyma [53]. Another carrier of Aβ in the blood is transthyretin (TTR), also known for transporting thyroid hormones and retinol [54,55]. Evidence indicates that TTR mitigates Aβ toxicity and may positively regulate the expression of LRP1. Moreover, TTR transports Aβ from the blood to the liver for further degradation [55].

Albumin is another key player involved in transporting amyloid-beta (Aβ) in the bloodstream. Albumin is the predominant protein in blood plasma and is involved in transporting various molecules. Biere et al. showed that almost 90% of plasma Aβ binds to albumin and about 5% to lipoproteins [50]. Kuo et al. reported that serum albumin binds over 95% of Aβ at a plasma concentration of 5 ng/mL [56]. The bulky nature of albumin interaction with Aβ may play an important role in Aβ transportation and clearance. Kim et al. investigated the possible correlation between albumin and Aβ accumulation in AD patients and suggested that reduced albumin levels could elevate the risk of AD by enhancing amyloid accumulation [57].

Recently, platelets have also been recognized as an important transporter of Aβ in the periphery. Numerous studies have demonstrated that platelets can bind and transport Aβ in the bloodstream, influencing its clearance and deposition. Furthermore, platelets are recognized as a significant source of peripheral Aβ [58,59,60,61]. In addition to serving as transport carriers for Aβ, platelets activated by Aβ undergoes a range of signaling pathways such as mitogen-activated protein kinases activation, cytoskeletal reorganization, abnormal ROS production, and others [62]. Activated platelets may be involved in cerebral and vascular AD pathology and can induce BBB dysfunction and Aβ influx transport [63].

Several groups have explored the idea that Aβ binds to RBCs. It was also hypothesized that plasma Aβ interacts with red blood cells (RBCs), disrupting their function in circulating blood [64,65]. Kiko et al. reported that Aβ_1–40_ and Aβ_1–42_ levels in human RBCs increase with age [66]. In RBCs, Aβ may bind to hemoglobin (Hb) and accumulate in vascular deposits, which is evidenced by the co-localization of Hb with amyloid plaques in post-mortem brains of AD patients [67,68]. Although it is evidenced that RBCs may have some role in Aβ transport, the extent of red blood cells’ contribution to its clearance remains under investigation. As a transporter, RBCs can bind Aβ, rapidly removing them from the blood to the liver for further clearance stages [69].

## 4. Peripheral Clearance of Aβ

There is strong evidence that the liver is one of the key players in the peripheral clearance of beta-amyloid [16,51,52]. Early studies have shown that the liver sequesters 40% of the total injected Aβ at 90 min post-injection, while kidneys contain only 5% of the peptide [51]. In a similar report, the liver absorbed about 65% of the intravenously injected radiolabeled Aβ_1–40_ and Aβ_1–42_, while the levels of labeled peptides in the kidneys and small intestine were less than 10%, and only trace amounts were found in other internal organs [52]. The authors of this study have proposed that peripheral Aβ is primarily absorbed by the liver, which then releases Aβ catabolites into the intestine through the bile ducts. Consequently, evidence exists that liver dysfunction can contribute to the accumulation of Aβ in the brain and AD progression [70,71,72].

In contrast to the brain, LRP1 is the primary molecule responsible for the hepatic uptake of Aβ from the blood, functioning as a clearance receptor for Aβ [29,73,74]. RAGE and P-gp are also involved in hepatic uptake of Aβ, but to a lesser extent than LRP1 [74]. In vitro investigation of Aβ hepatobiliary disposition in sandwich-cultured primary rat hepatocytes demonstrated that P-gp also plays an important role in the biliary excretion of Aβ breakdown products [74]. A recent study by Cheng et al. showed that chronic reduction of Aβ clearance in the liver through liver-specific LRP-1 knockdown in hepatocytes led to the accumulation of Aβ in the brain and cognitive impairments. Conversely, overexpression induced by transfection LRP-1 in a liver reduced Aβ deposition and cognitive impairments in APP/PS1 mice [16]. Sehgal et al. revealed that APP/PS1 mice exhibited decreased LRP1 and neprilysin levels in the liver and decreased plasma sLRP expression compared to the WT animals [75].

Monocytes are other key players in the peripheral and cerebral clearance of Aβ [76,77]. Although microglial cells are innate phagocytes that account for the majority of CNS mononuclear phagocytes in healthy brains, in some pathological conditions, including AD, peripheral monocytes migrate across the BBB and differentiate into activated macrophages within the brain parenchyma [78,79,80]. A study of phagocytosis of Aβ by microglial cells and macrophages in mice has shown that microglial cells can degrade fibrillar Aβ only to monomeric form. In contrast, macrophages not only degrade fibrils to monomers but also can destroy monomers themselves and release various-sized fragments of degraded Aβ [81]. In turn, dysfunction of the macrophage system is observed in AD patients, notably diminished ability of Aβ phagocytosis [77,82,83,84].

All three subsets of peripheral monocytes (classical, nonclassical, and intermediate) can phagocytize Aβ, however, the highest rate of Aβ uptake is typical for the intermediate monocyte subset [85,86]. A recent study by Huang et al. demonstrated that monocyte phagocytic activity correlates with surface levels of Aβ, and in AD patients, there is a 43% reduction in the percentage of monocytes capable of binding Aβ, along with a 26% decrease in Aβ levels on the surface of monocytes compared to the controls [86]. This study also examined cerebrospinal fluid monocytes, which were predominantly composed (90%) of intermediate monocytes (CD14+ CD16+) expressing CD68 and TREM2, resembling tissue macrophages and microglia. Such a high level of intermediate monocytes may be associated with the differentiation of classical monocytes into intermediate ones in conditions of elevated Aβ levels in cerebrospinal fluid. Huang et al. suggested that the primary Aβ phagocytosis by monocytes occurs in cerebrospinal fluid, perivascular spaces, and within the brain. The authors also demonstrated that monocytes are capable of migrating back from the brain into the bloodstream, potentially contributing to Aβ clearance across different compartments [86].

As an initial phagocytic stage, monocytes recognize Aβ through receptors on their surface such as toll-like receptors (TLR2, TLR4), triggering-receptor-expressed-on-myeloid-cells2 (TREM2), CD36, CD33, macrophage scavenger receptor 1 (SCARA1) and others. Thus, the impaired phagocytosis of Aβ by monocytes in AD might be attributed to the compromised levels of Aβ-recognizing receptors [87,88,89,90]. For example, La Rosa et al. reported that in AD patients, the amount of TREM2-expressing monocytes is reduced along with Aβ phagocytosis reduction [89]. Chen et al. reported that CD36, CD33, and SCARA1 receptor expression in AD monocytes is comparable to that in healthy individuals, but TLR2 receptor expression is lower in AD patients [85]. In contrast, Zhang et al. detected higher levels of mRNA and protein expression of TLR2 and TLR4 in blood mononuclear cells of AD patients [91].

Aβ recognition by monocytes may also be reduced in AD patients because of cystatin F protein. This protein is expressed in monocytes, lymphocytes, neutrophils, and brain microglial cells, and can directly interact with Aβ, preventing monocyte recognition [92]. It was reported that cystatin F expression increased in AD monocytes; moreover, in 5XFAD mice, high protein levels led to rapid cognitive impairment [92]. The cytoskeleton dysfunction is another critical factor that underlies the mechanics of all stages of phagocytosis. It remains unclear whether the cytoskeletal elements of monocytes in Alzheimer’s disease exhibit pathological abnormalities, however, in many cell types, Aβ disrupts F-actin organization [93,94,95]. Aβ may also disturb microtubule assembly in cells by blocking the binding domain of the microtubule-associated protein 1B [96].

Another potential cause of impaired peripheral Aβ degradation in monocytes may be attributed to dysfunctions in their digestive system involving vesicle trafficking and lysosomal function. The two main groups of lysosomal enzymes associated with AD are glycohydrolases and proteases (cathepsines). It has been reported that the activity of β-hexosaminidase, β-galactosidase, β-galactosylcerebrosidase, and β-glucuronidase were decreased in AD patients [97]. At the same time, the current understanding of cathepsin function impairment in AD monocytes remains incomplete and is a subject of ongoing debate: some studies have reported no significant differences in the expression of cathepsins S, D, B, and L between AD patients and healthy individuals [85,97], while others, such as the study by Tian et al., have found that cathepsin D expression is reduced in AD monocytes compared to normal subjects [98].

Thus, the role of the liver and monocytes in the peripheral clearance of Aβ is critical for maintaining homeostasis and preventing the accumulation of Aβ in the brain. Monocytes engage in the phagocytic clearance of Aβ, and evidence suggests that this process is impaired in AD patients, leading to increased Aβ accumulation and disease progression. In turn, there is compelling evidence supporting the role of liver diseases in the onset and exacerbation of Alzheimer’s disease. While the specific mechanisms by which liver disease affects the brain remain unclear, it appears that liver dysfunction may accelerate the progression of Alzheimer’s disease through impaired clearance of beta-amyloid. Addressing these dysfunctions could yield promising therapeutic strategies for mitigating AD progression.

## 5. Alzheimer’s Disease Risk Factors and Components of Peripheral Beta-Amyloid Catabolism System

The complexity of assessing the impact of Aβ peripheral clearance on AD lies in the fact that the impairment of Aβ clearance likely occurs over an extended period before the disease itself becomes apparent. Therefore, in this chapter we discuss the possible relationships between certain AD risk factors and peripheral Aβ clearance with the focus on non-genetic determinants that have a chronic impact.

Recent epidemiological studies have demonstrated that NAFLD (Non-Alcoholic Fatty-Liver Disease) is an important risk factor for Alzheimer’s disease [99,100,101]. In support of this notion, Peng and co-authors showed that Aβ clearance is impaired in rats with NAFLD [71]. They revealed that NAFLD in rats leads to reduced levels of LRP1 and Aβ in liver tissue, inversely proportional to Aβ concentrations in the brain, increased plasma Aβ_1–40_ and Aβ_1–42_ levels, and cognitive performance decline [71]. Monocyte activity is also suppressed in patients with NAFLD, which is accompanied by low expression of HLA-DR. This reduced functionality of monocytes may contribute to impaired immune responses and weakened clearance of harmful substances, such as beta-amyloid, in these individuals [102].

Several epidemiological studies also show a link between alcohol consumption and the onset of Alzheimer’s disease. These studies suggest that both heavy drinking and long-term alcohol abuse can increase the risk of cognitive decline and neurodegeneration, potentially contributing to the onset of Alzheimer’s disease [103]. Garcia et al. demonstrated that a four-week regimen of intragastric alcohol administration in C57BL/6J mice reduced liver LRP1 levels by 46% and doubled the levels of APP in the liver and brain [104]. The same authors demonstrated that a five-week exposure of APP/PS1 mice to alcohol significantly reduced LRP-1 expression in the liver, particularly in the most alcohol-damaged pericentral hepatocytes, and led to the accumulation of Aβ in the brain [105]. Alcohol consumption also negatively impacts monocyte activity, leading to a reduction in their phagocytic function, both in individuals with alcohol dependence and in non-alcoholics [106,107].

Conditions like metabolic syndrome, type 2 diabetes, and obesity are associated with systemic inflammation, insulin resistance, and increased risk of AD [108]. It has been shown that the LRP1 protein in the liver is also sensitive to glucose and insulin levels. For example, reduced expression of LRP1 in the liver was observed in diabetic OLETF rats, and an in vitro model of hyperglycemia (25 mM glucose in culture medium) suppressed LRP1 expression in HepG2 cells [109]. The relationship between insulin and the LRP1 protein is based on insulin’s ability to induce the translocation of hepatic LRP1 from intracellular vesicles to the plasma membrane, which enhances the uptake of LRP1-specific ligands. For instance, insulin treatment in wild-type mice increased hepatic LRP1 ligand uptake. In contrast, insulin-resistant obese mice with leptin deficiency (ob/ob) exhibited reduced LRP1 expression, and insulin administration did not lead to LRP1 ligand uptake in these mice [110]. The functions of blood monocytes are also significantly altered when insulin regulation is impaired [111].

As age is one of the most significant risk factors for AD, transcriptomic analysis of the liver in males revealed that LRP1 protein expression peaks before the age of 30, followed by a sharp decline as age increases [112]. An age-related decline in LRP1 expression and the hepatic uptake of Aβ_1–40_ has also been observed in rats, where a significant reduction in LRP1 levels was noted in 13-month-old rats compared to 7-week-old rats [29]. The uptake of Aβ_1–42_ by the overall monocyte population showed a clear age-related decline, with older individuals exhibiting lower levels of Aβ_1–42_ absorption. In particular, uptake by total monocytes and the CD14+ CD16− subset decreased rapidly in individuals aged 20–40, but this decline slowed after age 40. Similarly, the uptake by the CD14dim CD16+ subset showed a rapid reduction in individuals aged 40–60, with the rate of decline also becoming more gradual after 60. Notably, a non-linear dynamic in the expression of LRP1 and phagocytic activity has been observed in both the liver and monocytes, likely reflecting physiological processes associated with aging [113].

Thus, based on the above-mentioned findings, we hypothesize that the peripheral clearance of beta-amyloid is predominantly mediated by the liver and circulating blood monocytes, and dysfunctions in these systems, driven by certain external and internal factors result in chronic elevation of peripheral beta-amyloid levels accelerating its deposition in the brain and thereby increasing the risks of AD.

## 6. Aβ Degrading Enzymes

As evidenced by previous chapters, promoting the Aβ peripheral clearance might be an effective strategy to prevent the accumulation of β-amyloid peptide in the brain. Accordingly, by preventing the accumulation of Aβ peptide, it is possible to slow down the rate of development of Alzheimer’s disease. New views on the amyloid cascade hypothesis have led to the recognition of the dynamic equilibrium in which Aβ resides and the importance of enzymes involved in Aβ production and degradation in maintaining healthy Aβ levels. Recent evidence suggests that promoting the breakdown of Aβ, rather than inhibiting its synthesis, is an effective strategy to prevent the accumulation of β-amyloid peptide. Therefore, enzymes that degrade Aβ are valuable targets for the treatment of Alzheimer’s disease and are the focus of this chapter.

Neprilysin (NEP), also called membrane metalloendopeptidase (MME) or neutral endopeptidase, is one of the dominant proteolytic enzymes. It is a neutral endopeptidase belonging to the M13 peptidase family and encoded by the *MME* gene. Neprilysins are type II integral membrane proteins with an active site facing the extracellular environment [114]. Studies of NEP have shown its involvement in the pathophysiology of Alzheimer’s disease [115]. As an example, S. El-Amouri showed that human *(h)NEP* gene transfection to APP/PS1 transgenic mice decreased amyloidosis and associated pathogenetic changes in the brain [116].

Neprilysin-2 (NEP2) is a homolog of neprilysin, and it is assumed that NEP and NEP2 play different physiological roles in humans since the inhibitor binding and substrate specificity of NEP2 differ from those of NEP [117]. It has been shown that knockout of the NEP2 coding gene *MMEL1* (Membrane Metalloendopeptidase Like 1) caused an increase in Aβ levels in the hippocampus and diencephalic stem in a mouse AD model (5XFAD mice) [118]. This finding suggests that NEP2 may play a protective role against AD pathology.

Endothelin converting enzyme 1 (ECE-1) is another homolog of NEP, and it includes four isoforms, ECE1A, ECE1B, ECE1C, and ECE1D, differing in the N-terminal sequence. All four isoforms of ECE-1 are expressed on the cell surface and in endosomes. A study in mouse cell lines showed that overexpression of the ECE-1 enzyme significantly reduced the concentration of Aβ (up to 90%), while the inhibitor of ECE1 by phosphoramidon had the opposite effect [119]. It has been established that ECE1 cleaves Aβ at 3 sites with the formation of fragments Aβ_1–17_, Aβ _1–19_, and Aβ_20–40_ [120]. In contrast to NEP and NEP-like peptidases, which are most active at neutral pH, ECEs are mainly active at acidic pH, predominantly in acidic subcellular compartments. Therefore, ECEs primarily degrade Aβ at intracellular sites [121]. This point is significant because it shows that most of the Aβ degradation probably occurs before the monomer’s secretion into the extracellular space [122].

ECE-1 is synthesized as an endothelin precursor and is converted into mature biologically active endothelin-1 by a protein encoded by the ECE 1 gene [123,124]. It has been demonstrated that knockout of the ECE1 gene in animal models of AD leads to an increase in the level of Aβ [125]. In human studies, Western individuals homozygous for the C-338A polymorphism (AA) within the *ECE1* gene promoter region have been shown to be at reduced risk of developing late-onset Alzheimer’s disease (LOAD) [126]. These studies collectively suggest that the control of the expression level of this gene can be a preventive measure for Alzheimer’s disease. However, Zhao Jin and colleagues argue that the *ECE1* 338A allele is protective against LOAD in the Chinese population [127].

Endothelin-converting enzyme 2 (ECE2) is an important paralog of the ECE1. ECE2 is located in the membrane of the cytoplasmic vesicle. It activates metalloendopeptidase activity, participates in the conversion of large endothelin-1 to mature endothelin-1, and is involved in the processing of beta-amyloid and various neuroendocrine peptides [128]. Xinxin Liao et al. found that two mutations (R186C and F751S) located in the peptidase domain of the ECE2 protein significantly impair the enzymatic activity of ECE2 in degrading Aβ. However, overexpression of wild-type ECE2 in the hippocampus reduces amyloid load and plaque formation and improves learning and memory deficits in a mouse model of AD [129].

Another important enzyme involved in the degradation of Aβ is the angiotensin-converting enzyme I (ACE). This enzyme causes sodium retention in the kidneys and promotes the conversion of angiotensin I into a powerful vasopressor, angiotensin II, which constricts blood vessels and causes an increase in blood pressure [130]. ACE prevents aggregation and plaque formation of amyloid-beta by accelerating the degradation Aβ_1–40_ and Aβ_1–42_ peptides [131]. Mutations in the gene encoding ACE are associated with a wide range of diseases, including the pathophysiology of the entire cardiovascular system, renal failure, psoriasis, and Alzheimer’s disease. Jacob S Elkins et al. found that allele I of the *ACE* D/I polymorphism is associated with an increased risk of developing late-onset AD. The risk of AD associated with this allele was higher among Asians compared with the risk among and in younger cases (mean age 65 to 74 years) compared with the risk in older cases [132].

Another important group of Aβ-degrading proteases capable of degrading monomeric and fibrillar Aβ structures are matrix metalloproteinases (MMPs) [133]. Matrix metalloproteinases are zinc-dependent endopeptidases belonging to the M10A peptidase family, and they are the main proteases involved in the degradation of the intracellular matrix [134]. Ke-Jie Yin et al. have demonstrated that MMPs are activated in astrocytes adjacent to amyloid deposits [135]. A study by Deb and Gottschall showed that MMPs can be activated by degradation and other pathological damage to Aβ [136].

Membrane palmitoylated protein 2 (MPP2) is a member of the M10A family, and *MPP2* (MAGUK P55 framework protein 2) is a gene encoding the protein [137]. A case-control study was carried out to elucidate the association of *MMP2* gene candidate polymorphisms with the susceptibility to Alzheimer’s disease (AD) in the Chinese Han population [138]. The study reported that *MMP2* gene rs243866 and rs243865 polymorphisms were closely associated with the onset age of AD. The presence of rs243866 AA genotype emerged as a crucial predictor of AD risk.

Another matrix metalloproteinase that also affects beta-amyloid degradation is CD147/EMMPRIN [139]. It was found that the knockdown of CD147 leads to a significant increase in the production of Aβ peptides [140]. Furthermore, turning off this gene in mice leads to memory loss and disorientation, which is characteristic of Alzheimer’s disease. However, the mechanisms by which CD147 regulates Aβ levels remain unclear. CD147 may be involved in neuron-glial interactions and regulate neuroinflammation processes. It was found that CD147 affects the activity of the gamma-secretase complex, which carries out intramembrane endoproteolysis of the APP. Deletion of the gene encoding CD147 in mice has resulted in various neurological disorders, including severe neurodevelopmental defects [141,142].

IDE (Insulin Degrading Enzyme) is a zinc metallopeptidase belonging to the M16 peptidase family. The preferential affinity of this enzyme for insulin leads to insulin-mediated inhibition of the degradation of other peptides such as glucagon, bradykinin, natriuretics, kallidin, and others [143,144,145,146]. IDE also plays a significant role in the degradation and clearance of amyloidogenic peptides derived from APP secreted by neurons and microglia [147], and a deficiency in the function of this protein is associated with Alzheimer’s disease. Nicole Schupf et al. demonstrated that IDE activity is increased in amyloid plaques, and lower IDE expression is found in the hippocampal brains of older LOAD individuals who are APOE ε4-positive, as well as in individuals with mild cognitive impairment, who are at most significant risk for LOAD [148]. Lower IDE expression was also correlated with higher cellular Aβ_1–42_ levels associated with PSEN1 mutations in cellular models.

S. Vepsäläinen et al. showed that polymorphisms in the genes encoding NEP and IDE individually influence susceptibility to AD among the population of Finland. The result of the study was that the combination of risk genotypes for the *NEP* and *IDE* genes leads to a higher susceptibility to AD. Individuals with a combination of *NEP* and *IDE* risk genotypes had a threefold higher susceptibility to AD compared with individuals not carrying these genotypes. Although no significant interaction between *NEP* and *IDE* genes was observed, these data suggest that NEP and IDE exhibit an additive risk effect in AD [149].

Overall, these collective findings from various studies highlight the significant role of enzymes involved in Aβ degradation in the pathology of Alzheimer’s disease. Genetic polymorphisms affecting these enzymes further illustrate the complexity of AD risk. They suggest that targeting the regulation and expression of these genes and their associated pathways could offer new avenues for preventing or attenuating the progression of Alzheimer’s disease, and continued research in this area is crucial for developing effective interventions and understanding the genetic underpinnings of AD.

## 7. Targeting the Regulation of Beta-Amyloid Clearance for Clinical Use

Anti-Aβ immunotherapy is currently the most studied approach for improving Aβ clearance [150]. Advancements in passive anti-amyloid immunotherapy research involve the discovery of antibodies that promote microglial activation, catalyze amyloid disaggregation, and enhance the clearance of Aβ from CSF to plasma, thereby reducing the neurotoxic effects of Aβ. The FDA has approved three antibody-based drugs for clinical use in Alzheimer’s disease: Aducanumab, Lecanemab, and Donanemab [151]. These drugs are designed to slow or prevent disease progression by enhancing the clearance of Aβ. Despite their limited efficacy and drawbacks, such as the frequency of administration and effectiveness primarily in the early stages of the disease, the approval of these drugs suggests that targeting Aβ clearance remains a promising therapeutic strategy. Given its critical role in the pathological cascade of Alzheimer’s disease, any step in the Aβ clearance process could potentially serve as a viable target for therapeutic intervention.

Targeting LRP1 associated with peripheral clearance of beta-amyloid is another promising molecular target for Alzheimer’s disease therapy. Although there are currently no successful clinical trials targeting LRP1, several in vivo studies have shown that plant extracts and a number of well-known medical drugs can enhance LRP1 expression in tissues [75,109,152,153]. Sehgal et al. demonstrated that a 30-day treatment with *Withania somnifera* (WS) extract increased plasma sLRP1 levels and enhanced LRP1 expression, reducing cerebral Aβ levels in mice. Suppression of hepatic LRP1 resulted in increased Aβ levels in the brain and decreased Aβ levels in the plasma, while the inhibition of hepatic neprilysin did not affect Aβ levels in the brain and plasma [75]. The well-known anti-diabetic drug of the thiazolidinedione class, rosiglitazone, can also activate LRP1 in the liver. It has been shown that rosiglitazone increases LRP1 expression through a PPARγ-dependent regulatory mechanism. In vivo studies on diabetic OLETF rats demonstrated that five weeks of rosiglitazone treatment significantly enhanced LRP1 expression in the liver [109]. Although the results of clinical trials of rosiglitazone did not show significant efficacy in the treatment of Alzheimer’s disease, some researchers suggest that its effectiveness can be improved through targeted delivery using nanoparticles [154,155].

The next class of drugs capable of activating LRP1 may include statins. Moon and co-authors have demonstrated that atorvastatin regulates hepatic LRP1 expression through sterol response element-binding protein–2 (SREBP-2). They found that six weeks of treatment with the drug could induce LRP1 expression in the liver of healthy and diabetic animals [152]. In another study, simvastatin in the composition of nanoparticles activated LRP1 expression in the brain of ICR mice [153]. A recent systematic review and meta-analysis on the relationship between statin use and the risk of dementia or Alzheimer’s disease showed that statin reduces the risk of developing dementia [156]. Thus, the regulation of LRP1 in the liver is a promising target for Alzheimer’s disease therapy, and LRP1 activators, such as plant extracts, anti-diabetic drugs, and statins, may form the basis for further development of new therapeutic approaches.

Other potential therapeutic strategies could be focused on enhancing the efflux and influx of Aβ through transporters, such as LRP and RAGE receptors, and the digestion of Aβ by monocytes’ phagocytosis, which serves as the bloodstream’s first clearance line. Additionally, Aβ-degrading enzymes offer another approach to regulating Aβ clearance, which could involve either inducing the production or activity of these enzymes within the body or administering them as a medication. While both strategies hold significant potential, there is skepticism about using enzymes in therapy due to their low specificity. Although many proteases are involved in Aβ degradation, no studies have examined the use of these enzymes for Alzheimer’s treatment yet.

## 8. Conclusions

**Figure 2:**
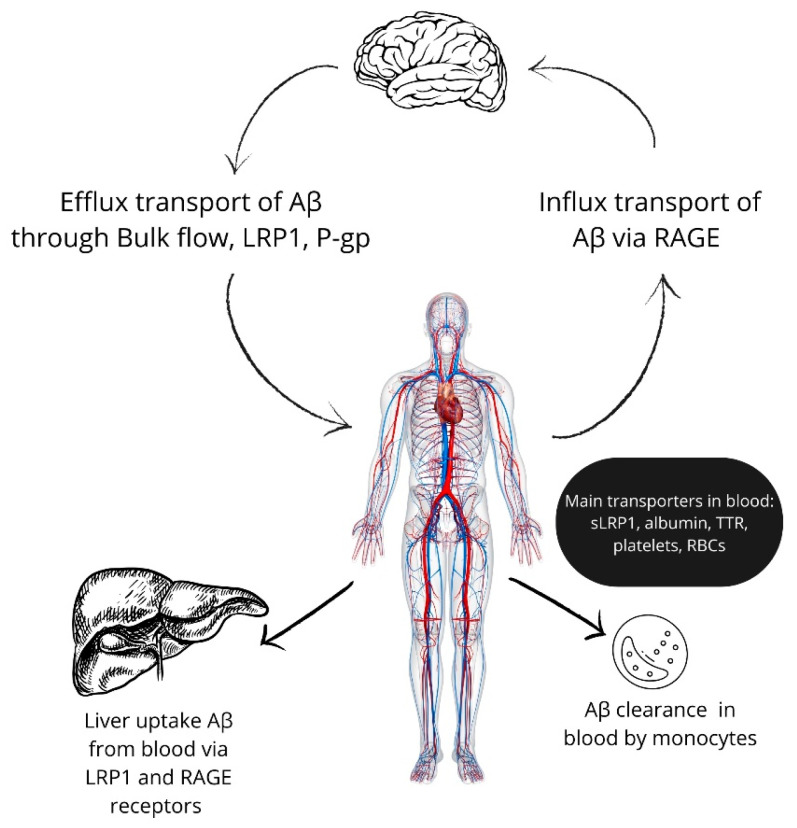
Schematic pathways of peripheral metabolism of Aβ.

In summary, the peripheral clearance of Aβ is not a linear or isolated process, but involves a dynamic and interconnected network of organs, cells, enzymes, and receptors (Figure 2). A major limitation of most studies on beta-amyloid clearance is the tendency to examine individual components of the clearance system in isolation without considering the complex interactions between key elements. Disruptions in one part of the system—whether through age-related changes, insulin resistance, or alcohol consumption—can have cascading effects on the entire clearance process. The failure to study these elements as part of a comprehensive system limits our understanding of how peripheral factors, such as liver function or monocyte activity, interact with brain processes and contribute to Alzheimer’s disease progression. A more holistic approach, integrating the various players in this system, is necessary to fully grasp the complexity of beta-amyloid clearance and its role in Alzheimer’s disease development.

## Data Availability

Not applicable.
